# GLP-1 action in the mouse bed nucleus of the stria terminalis

**DOI:** 10.1016/j.neuropharm.2017.12.007

**Published:** 2018-03-15

**Authors:** Diana L. Williams, Nicole A. Lilly, Ian J. Edwards, Pallas Yao, James E. Richards, Stefan Trapp

**Affiliations:** a Psychology Department & Program in Neuroscience, Florida State University, USA; b Centre for Cardiovascular and Metabolic Neuroscience, Department of Neuroscience, Physiology & Pharmacology, University College London, London, WC1E 6BT, UK

**Keywords:** Glucagon-like peptide-1 receptor, Electrophysiology, Channelrhodopsin, Preproglucagon, BNST, PPG, Food intake, Stress, 3V, third ventricle, AAV, adeno-associated virus, ACSF, artificial cerebrospinal fluid, Arc, arcuate nucleus, BNST, bed nucleus of the stria terminalis, CeA, central amygdaloid nucleus, DMH, dorsomedial hypothalamus, Ex9, exendin (9-39), GLP-1, glucagon-like peptide-1, GLP-1R, GLP-1 receptor, HFD, high fat diet, LS, lateral septum, LV, lateral ventricle, ME, median eminence, MeA, medial amygdaloid nucleus, MM, medial mammillary nucleus, MnPO, median preoptic nucleus, opt, optic tract, MPA, medial preoptic area, MS, medial septum, NAc, nucleus accumbens, NTS, nucleus of the solitary tract, PB, phosphate buffer, PFA, paraformaldehyde, Pir, piriform cortex, PPG, preproglucagon, PVN, paraventricular nucleus, SHi, septohippocampal nucleus, TTX, tetrodotoxin, VMN, ventromedial nucleus of the hypothalamus, VP, ventral pallidum, VTA, ventral tegmental area

## Abstract

Glucagon-like peptide-1 (GLP-1) injected into the brain reduces food intake. Similarly, activation of preproglucagon (PPG) cells in the hindbrain which synthesize GLP-1, reduces food intake. However, it is far from clear whether this happens because of satiety, nausea, reduced reward, or even stress. Here we explore the role of the bed nucleus of the stria terminalis (BNST), an area involved in feeding control as well as stress responses, in GLP-1 responses.

Using cre-expressing mice we visualized projections of NTS PPG neurons and GLP-1R-expressing BNST cells with AAV-driven Channelrhodopsin-YFP expression. The BNST displayed many varicose YFP+ PPG axons in the ventral and less in the dorsal regions. Mice which express RFP in GLP-1R neurons had RFP+ cells throughout the BNST with the highest density in the dorsal part, suggesting that PPG neuron-derived GLP-1 acts in the BNST. Indeed, injection of GLP-1 into the BNST reduced chow intake during the dark phase, whereas injection of the GLP-1 receptor antagonist Ex9 increased feeding. BNST-specific GLP-1-induced food suppression was less effective in mice on high fat (HF, 60%) diet, and Ex9 had no effect. Restraint stress-induced hypophagia was attenuated by BNST Ex9 treatment, further supporting a role for endogenous brain GLP-1. Finally, whole-cell patch clamp recordings of RFP+ BNST neurons demonstrated that GLP-1 elicited either a depolarizing or hyperpolarizing reversible response that was of opposite polarity to that under dopamine.

Our data support a physiological role for BNST GLP-1R in feeding, and suggest complex cellular responses to GLP-1 in this nucleus.

## Highlights

• NTS PPG neurons project strongly to the BNST.
• Exogenous or endogenous GLP-1 acting in the BNST potently reduces food intake.
• Stress-induced hypophagia is attenuated by GLP-1 receptor blockade in the BNST.
• Within the BNST, GLP-1 elicits both excitatory and inhibitory electrical responses.
• GLP-1 likely acts on both orexigenic and anorexigenic efferent pathways from the BNST.

## Graphical abstract

**Figure:**
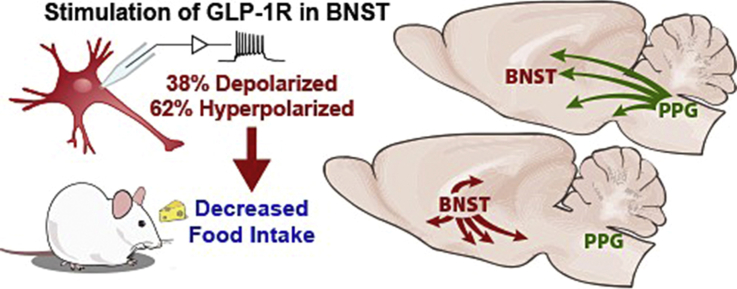

## 1 Introduction

Glucagon-like peptide 1 (GLP-1) expressing preproglucagon (PPG) neurons of the nucleus of the solitary tract (NTS) are widely considered to play an important role in the control of food intake, body weight, and responses to stress (Gaykema et al., 2017, Holt and Trapp, 2016, Maniscalco et al., 2015, Trapp and Cork, 2015). These neurons project throughout the brain to many regions in which GLP-1 receptors (GLP-1R) are expressed (Cork et al., 2015, Llewellyn-Smith et al., 2011). A variety of behavioral effects of GLP-1R activation can be elicited by selectively targeting some of these locations (Alhadeff et al., 2012, Alhadeff et al., 2014, Dossat et al., 2013, Richard et al., 2014, Shirazi et al., 2013, Williams, 2014).

Most investigations of the behavioral effects of brain GLP-1R manipulations have been performed in rat, and data from studies in mouse suggest species differences which in some cases have called into question whether neuronal GLP-1R contributes to the physiological control of feeding (Sisley et al., 2014). However, hypothalamic knockdown of GLP-1R in the mouse increases food intake (Burmeister et al., 2017), although the specific cell populations involved in this effect are unclear. Increased feeding was not recapitulated by knockdown of GLP-1R in SIM1-expressing neurons of the paraventricular nucleus (PVN) or POMC neurons of the arcuate nucleus (ARC), however, the loss of GLP-1Rs in PVN neurons does attenuate a variety of physiological and behavioral responses to both acute and chronic stress (Ghosal et al., 2017).

A number of recent studies targeting mesolimbic nuclei in rat have demonstrated a role for GLP-1R in food intake. In rats, activation of GLP-1R in major centers of the mesolimbic dopamine pathway involved in reward (nucleus accumbens (NAc) and ventral tegmental area (VTA)), reduces eating motivation, food intake, and (in the NAc) palatability (Alhadeff et al., 2012, Dossat et al., 2011, Dossat et al., 2013). These studies suggest a role for GLP-1 in hedonic feeding, but the details of GLP-1 innervation and GLP-1R expression in mesolimbic areas is poorly understood. The availability of mouse strains expressing Cre recombinase under the PPG or the GLP-1R promoters enables further investigation (Parker et al., 2012, Richards et al., 2014).

Here we present analysis of projections from NTS PPG neurons to the mesolimbic system as well as identifying bed nucleus of the stria terminalis (BNST) GLP-1R-derived projections using Cre-dependent virally assisted neuroanatomical tracing. We demonstrate that the BNST, an area known to be involved in feeding and stress responses, receives substantial innervation from NTS PPG neurons, and that GLP-1R expressing neurons of the BNST project widely to other brain areas. We then establish food intake suppressive effects when BNST GLP-1Rs are stimulated both exogenously and endogenously. Finally, we show that individual GLP-1R-expressing neurons in the BNST respond to GLP-1 stimulation with either excitation or inhibition of electrical activity.

## 2 Material and methods

### 2.1 Animals

Naïve male C57Bl6J mice (Jackson Laboratories) were used in the behavioral experiments. Studies examining distribution of GLP-1 neuronal axons utilized mGLU-124 Venus expressing mice (Reimann et al., 2008) (referred to as YFP-PPG) and mGlu-Cre:tdRFP mice (Parker et al., 2012). Mice that express Cre-recombinase under the control of the *Glp1r* promoter were crossed with the ROSA26-tdRFP reporter strain to yield GLP-1R-Cre:tdRFP progeny (Cork et al., 2015, Richards et al., 2014). Animals were maintained on a 12:12 light:dark schedule in a temperature controlled vivarium with *ad lib* access to water and standard chow unless otherwise specified below. All experiments performed in the UK were carried out in accordance with the UK Animals (Scientific Procedures) Act, 1986, with appropriate ethical approval. Studies performed in the US were approved by the Florida State University Institutional Animal Care and Use Committee and conformed to the standard of the National Research Council Guide for the Care and Use of Laboratory Animals, 2011.

### 2.2 Anatomical analysis

#### 2.2.1 Fourth generation neuroanatomical tracing

Selective fluorescent protein expression by cre-dependent adeno-associated virus (AAV) injection, also termed ‘fourth generation neuroanatomical tracing’ (Wouterlood et al., 2014) was employed to verify that PPG axons identified in the BNST originate from NTS PPG neurons and to identify projection targets of BNST neurons expressing GLP1-R. AAV particles were produced as described by Murray et al. (2011). Briefly, pAAV-EF1a-double floxed-hChR2(H134R)-EYFP-WPRE-HGHpA (gift from Karl Deisseroth; Addgene plasmid # 20298), AAV1 (pH21), AAV2 (pRV1) helper plasmids and the adenovirus helper plasmid pFΔ6 were co-transfected in HEK293 cells to produce AAV virions containing a 1:1 ratio of type 1 and 2 capsid proteins. 64 h post-transfection, cells were harvested and AAVs were purified from lysates using 1 ml HiTrap heparin columns (Sigma). Eluted virions were concentrated using Amicon Ultra-4 centrifugal filter devices (Millipore UFC810024).

Adult GLP-1R-CRE:tdRFP (female; n = 3) and mGlu-Cre:tdRFP (male; n = 3) mice were anaesthetised by intramuscular injection of ketamine (75 mg/kg) and medetomidine (0.5 mg/kg) to induce deep anaesthesia which was confirmed by the absence of a pedal withdrawal reflex. The mGlu-Cre:tdRFP mice were placed in a stereotaxic frame with their head tilted downwards to expose the neck. An incision was made along the midline and neck muscles were parted to expose the dura mater, which was ruptured, to facilitate bilateral injection into the NTS with 200 nl of AAV encoding ChR2:EYFP infused at 50 nl per min. The injection coordinates were (from calamus scriptorius): 0.1 mm rostral, 0.5 mm lateral, 0.35 mm ventral to surface. Anaesthesia was reversed using Antisedan (5 mg/kg) and buprenorphine analgesia (0.05 mg/kg per day; s.c. for 48 h) and s.c saline solution (150 μl per 30 g bodyweight) were provided.

GLP-1R-Cre:tdRFP mice, fixed in a stereotaxic frame, had cranial windows drilled to allow injections with 150 nl (50 nl/min) of AAV encoding ChR2:EYFP in each BNST location in both hemispheres to target GLP-1R neurons. The coordinates used to target the BNST were 0.25 mm rostral, 0.8 mm lateral, 4.25 and 4.75 mm ventral to the surface of the skull from Bregma. This ensured virus reached the BNST both dorsal and ventral of the anterior commissure. Anaesthesia was reversed and analgesia provided as described above. 21 days later, animals were transcardially perfuse-fixed as described below. Successful targeting was confirmed by the observation of somatic expression of ChR2 in tdRFP-positive cell bodies in the target area.

#### 2.2.2 Immunofluorescence

Mice, either naïve or three weeks after stereotaxic injection of AAV, were anaesthetised as described above, heparinized (50 units bolus) and transcardially perfused with 50 ml 0.1 M phosphate buffer (PB) followed by 50 ml of fixative (4% PFA in 0.1 M PB). Brains were extracted and postfixed overnight at 4 °C in fixative, then cryoprotected with 30% w/v sucrose in 0.1 M PB. Brains were sectioned coronally at 30 μm thickness on a cryostat (OTF cryostat; Bright Instruments Ltd.; Luton; UK).

Sections were blocked with 0.1% Triton X-100 and 10% normal goat serum diluted in 0.1 M PB for 1 h at room temperature, and were then incubated overnight at 4 °C with chicken anti-GFP (Catalogue #AB13970, lot #623923; Abcam, Cambridge MA, USA; YFP-PPG brains) or rabbit anti-DsRED (Clontec #632496; GLP-1R-Cre-tdRFP brains) (both 1:1000) in blocking solution. Subsequently, sections were washed 3 times for 5 min in 0.1 M PB at room temperature, followed by incubation with a fluorescently labeled secondary antibody, either goat anti-chicken IgG Alexa Fluor 488 (Catalogue# A-11039, Invitrogen) or sheep anti-rabbit IgG CY-3 (Catalogue# C2306, Sigma Aldrich, St Louis MO, USA) (both 1:500), in blocking solution for 2 h. Sections were washed again 3 times for 5 min in 0.1 M PB at room temperature and placed onto polysine-coated slides. Vectashield mounting medium (Vector Labs) was dispersed over the air dried sections and overlayed with a coverslip. Antibody specificity was confirmed by lack of positive staining in sections from naïve tissue. These primary antibodies have been well characterized in previous studies in our laboratory, demonstrating that the anti-GFP antibody recognizes EYFP and the anti-DsRED antibody recognizes tdRFP (Cork et al., 2015, Llewellyn-Smith et al., 2011). Fluorescent cells were visualized using a Leica epifluorescence microscope (Leica DMRB, Leica Microsystems, Milton Keynes, UK) and images captured with a RetigaTM 3000 CCD camera (QImaging, Surrey, Canada). Images were merged using ImageJ software and composited using Microsoft ICE software.

### 2.3 Feeding experiments

Male C57BL6 mice weighing an average of 22 g at the time of surgery were individually housed for these experiments. Under isoflurane anaesthesia, most mice were stereotaxically implanted with unilateral guide cannulas (26 G, Plastics One, Roanoke, VA, USA) at 0.5 mm anterior to Bregma, 0.7 mm lateral to midline, and 1.75 mm ventral to skull surface. Injectors (33 G) extended below the end of the guide by 3.25 mm to target the BNST. One group received unilateral guide cannulas to allow targeting of the NAc, implanted at 1.0 mm anterior to Bregma, 1.0 mm lateral to midline, and 2.0 mm ventral to skull surface, for use with injectors extending 2.75 mm below the end of the guide cannula. Cannula placements were verified histologically after behavioral experiments were completed, and n numbers noted here represent only mice with correct placements (hit rate = 85%). Recovered mice were habituated to handling and the injection procedure for at least 1 week before experiments. Food intake during the 20-h period post habituation saline injection did not differ from intake measured on non-injection days.

#### 2.3.1 Dose responses

This series of experiments utilized within-subjects designs, such that mice in a given dose response study received each injection condition in counterbalanced order separated by 72–96 h. On experiment days, chow was removed 3 h before dark onset and mice (n = 15, mean ± SEM body weight 25.9 ± 0.1 g at start of the experiment) were injected with 0.5 μl saline or either 0.1, 0.3, or 1.0 μg of GLP-1 (American Peptide, Vista, CA, USA) approximately 30 min before dark onset. Another group of mice (n = 16, mean ± SEM body weight 24.7 ± 0.1 g at start of the experiment) received intra-BNST injections of saline, 3 or 10 μg of the GLP-1R antagonist exendin (9-39) (Ex9), (American Peptide, Vista, CA, USA). These doses of GLP-1 and Ex9 were previously determined to be subthreshold for effect when delivered to the lateral ventricle of mice (unpublished preliminary data). Food was returned immediately before dark onset, and consumption was measured at 1, 2, 4, and 22 h. Additional groups of mice were maintained on a diet containing 60% fat (HFD; D12492, Research Diets, New Brunswick, NJ, USA) instead of chow for 2 weeks prior to injections. These groups received the same treatment as described above, either, saline and GLP-1 (n = 11, mean ± SEM body weight 28.8 ± 0.3 g at start of the experiment), or saline and Ex9 (n = 9, mean ± SEM body weight 29.4 ± 0.3 g at start of the experiment). In all experiments, body weight was recorded daily, but because there were no effects from drug treatment on weight in any study, these data are not shown.

Because the NAc, in close proximity to the BNST, has been implicated in feeding effects of GLP-1 in the rat (Alhadeff et al., 2012, Dossat et al., 2011), we examined the effect of the same doses of Ex9 delivered to the NAc, using the within-subjects design as described above. Naïve chow-maintained mice (n = 12, mean ± SEM body weight 23.5 ± 0.1 g at start of the experiment) were habituated to the injection procedures as described above, then they were injected with saline, 3 or 10 μg of Ex9 approximately 30 min before dark onset. Food was returned immediately before dark onset, and intake was measured 1, 2 and 22 h later. Drug treatments occurred in counterbalanced order separated by 48–72 h, with all mice receiving all conditions.

#### 2.3.2 Stress-induced hypophagia

Mice maintained on chow diet received either intra-BNST saline (n = 5, mean ± SEM body weight 27.8 ± 0.4 g at the start of the experiment) or intra-BNST Ex9 (3 μg dose, n = 5, mean ± SEM body weight 26.9 ± 0.2 g at the start of the experiment). On experiment days, food was removed 4 h before dark onset, and mice were injected with saline or Ex9 at 45 min before dark. On no-stress control days, mice were then handled briefly and placed back into their home cage. On stress days, mice were placed into a plastic DecapiCone (Braintree Scientific, Braintree, MA, USA) and restrained for 30 min. At the end of the 30 min restraint stress period, they were returned to their home cages. Pre-weighed food was returned immediately before dark onset, and intake was measured 1, 2, 4, and 22 h later. Each mouse received both a no-stress control and stress treatment, so this experiment had a mixed between/within-subjects design. Body weight was recorded daily, but as no effect from any treatment was observed, these data are not shown.

### 2.4 Electrophysiological recordings

All electrophysiological recordings were performed on adult transgenic GLP-1R-Cre:tdRFP mice of either sex. Altogether, recordings were obtained from 10 male and 12 female mice. Animals were culled by isoflurane overdose and the forebrain rapidly excised and transferred into ice-cold low Na^+^/low Ca^2+^ artificial cerebrospinal fluid (ACSF) of the following composition (in mmol/l): 200 sucrose, 2.5 KCl, 28 NaHCO_3_, 1.25 NaH_2_PO_4_, 3 pyruvate 7 MgCl_2_, 0.5 CaCl_2_, 7 glucose (pH 7.4). Coronal (200 μm) brain slices containing the bed nucleus of the stria terminalis (BNST) were sectioned on a vibratome (Campden Instruments) and kept at 34 °C in ACSF of the following composition (in mmol/l): 118 NaCl, 3 KCl, 25 NaHCO_3_, 1 MgCl_2_, 2 CaCl_2_, 10 glucose (pH 7.4). Experimental solutions were constantly bubbled with 95% O_2_/5% CO_2_.

Recombinant human GLP-1 (7-37) was obtained from Peprotech (London, UK), Tetrodotoxin (TTX) from Cayman Chemicals, 6,7-Dinitroquinoxaline-2,3-dione (DNQX) and (2R)-amino-5-phosphonovaleric acid (APV) from Tocris Bioscience (Bristol, UK), and dopamine from Sigma. DNQX was prepared as a stock solution in DMSO. All other stock solutions were prepared in H_2_O, and diluted at least 1000× when added to the ACSF.

All patch-clamp recordings were carried out in ACSF at 28–32 °C using an EPC-9 amplifier and Pulse/Pulsefit software (HEKA-Electronics, Germany), as described previously (Cork et al., 2015). Recording electrodes (3–8 MOhm) were filled with ‘low Cl^−^’ pipette solution composed of (in mM): 120 K-gluconate, 5 HEPES, 5 BAPTA, 1 NaOH, 1 MgCl_2_, 1 CaCl_2_ and 2 K_2_ATP; pH 7.2. The junction potential of −10 mV was corrected for offline. All figures display corrected potentials. Both whole-cell and loose-patch recordings were performed with the same solution. Holding potentials were as indicated in the results. Individual BNST cells expressing GLP-1 receptors were identified by their tdRFP fluorescence. Action potential frequency in individual recordings was determined as instantaneous frequency obtained with the Strathclyde Electrophysiology Software package (WinEDR/WinWCP; J. Dempster, University of Strathclyde, Glasgow, UK). Data are given as mean ± one S.E.M.

### 2.5 Statistical analysis

Effects of GLP-1 and Ex9 on food intake were evaluated via one-way within-subjects ANOVA, with Holm-Bonferroni post-hoc pairwise comparisons. The effects of restraint stress and intra-BNST Ex9 on feeding were assessed by mixed-design two-way ANOVA, with Holm-Bonferroni post-hoc pairwise comparisons. For analysis of electrophysiological data it was first determined whether or not a cell responded to a drug. An unpaired *t*-test was used to compare the instantaneous firing rate over 2 min in either the presence or absence of the drug. A value of p < 0.05 was taken as a positive response. Effects of drugs on mean membrane potential and mean firing rate were then evaluated in cells that had been determined as responsive, using a paired T-test, unless stated otherwise. P values < 0.05 (*) and <0.01 (**) were taken to indicate that the data were significantly different.

## 3 Results

### 3.1 Anatomy of the central GLP-1 system in the mesolimbic system

#### 3.1.1 Distribution of PPG axons

Analysis of three YFP-PPG brains revealed intense YFP-immunoreactivity throughout the cytoplasm of PPG neurons, allowing clear distinction of axons, cell bodies and dendrites throughout the brain. Densest innervation with PPG axons was identified in the hypothalamic nuclei of PVN, dorsomedial hypothalamus (DMH), ARC and periventricular nucleus (Pe) as previously reported (Llewellyn-Smith et al., 2011).

**Fig. 1:**
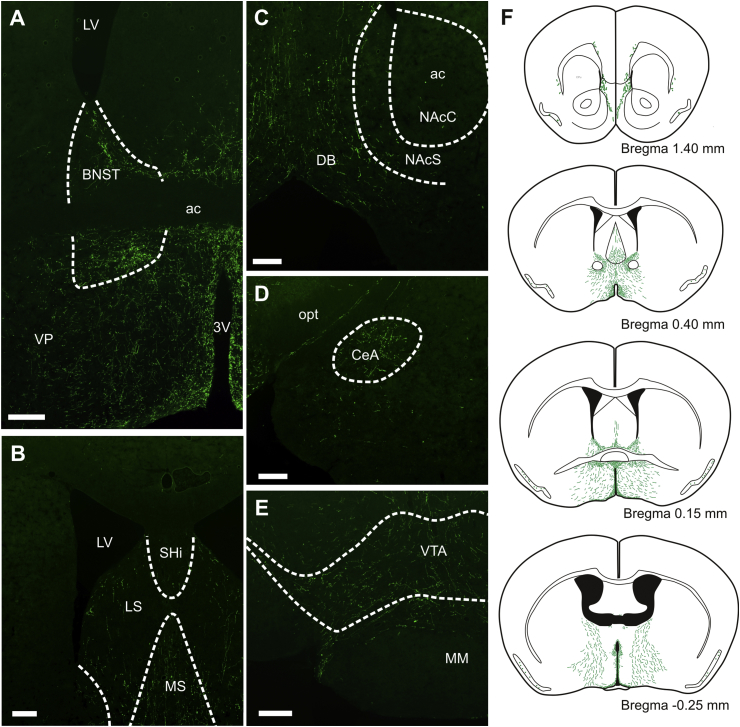
Projections from YFP-PPG neurons in the brainstem to key components of the limbic system. EYFP is strongly expressed throughout the dendritic and axonal arbours of YFP-PPG neurons in the brainstem. Varicose axonal projections can be observed throughout the bed nucleus of the stria terminalis (BNST; dorsal and ventral to the anterior commissure (ac)) (**A**), the medial (MS) and lateral septum (LS; **B**), the diagonal band of Broca (DB) but not the nucleus accumbens core (NAcC) or shell (NAcS; **C**), the central subnucleus of the aymgdala (CeA; **D**) and throughout the ventral tegmental area (VTA; **E**). The distribution of fibres throughout the mesolimbic system can be seen in the line diagrams (**F**), of particular note are the clustering of fibres around all rostro-caudal aspects of the BNST. All scale bars equal 200 μm. 3 V: third ventricle; LV: lateral ventricle; MM: medial mammillary nucleus; opt: optic tract; SHi: septohippocampal nucleus; VP: ventral pallidum.

Substantial numbers of PPG axons were identified in mesolimbic areas of the forebrain that were not analyzed in detail previously. Within these areas, the highest density of PPG axons was seen in the BNST (Fig. 1A), at approximately 0.14 mm anterior from Bregma at the anterior commissure. The medial division received PPG innervation of almost comparable density to the hypothalamic nuclei, and many PPG axons were found throughout the BNST. The lateral division of BNST received moderate to high innervation, which extended into the ventral pallidum (VP) (Fig. 1A).

Rostral to this, PPG axons at low to moderate density were identified in the dorsal and ventral parts of the lateral septum (LS) proximal to the lateral ventricles, the medial septum (MS) and occasionally the septohippocampal nucleus (SHi) (Fig. 1B and C). Rarely were PPG axons seen in the rostral portion of the nucleus accumbens (NAC; Fig. 1F); it was devoid of PPG axons in both the core (NAcC) and shell (NAcS). More caudally, a moderate density of PPG axons was observed along the midline in the diagonal band of Broca (DB) with some PPG axon terminals seen to cross the border into the NAcS and very occasionally into the NAcC (Fig. 1C). A few axons were identified in the cingulate cortex and claustrum (not shown). The central nucleus (CeM), medial nucleus (MeA) and extended amygdala (EA) revealed a low to moderate density of PPG axon innervation (Fig. 1D) and the VTA displayed a moderate density of PPG axons (Fig. 1E).

#### 3.1.2 NTS-PPG neuron projections to the BNST

**Fig. 2:**
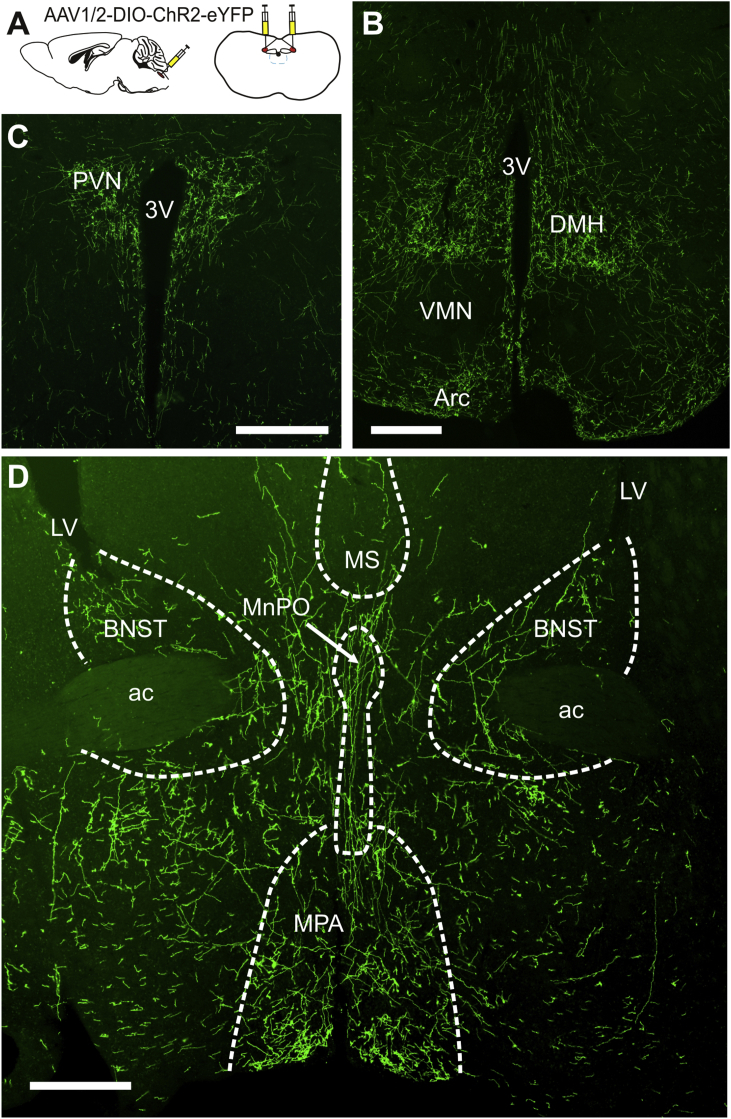
Virally mediated cre-dependent tracing of NTS PPG projections reveals strongly targeting of the BNST. Bilateral stereotaxic injections of AAV encoding a Cre-dependent Channel Rhodopsin 2 EYFP fusion protein were targeted to the NTS (**A**). Projections from the NTS PPG neurons could be observed within known target areas including the dorsomedial hypothalamus (DMH; **B**) and paraventricular nucleus (PVN; **C**). Strong axonal projections with varicose terminal sprays could be observed within the BNST dorsal and ventral to the anterior commissure (ac; **D**) All scale bars equal 200 μm. 3 V: third ventricle; Arc: arcuate nucleus; LV: lateral ventricle; MnPO: median preoptic nucleus; MPA: medial preoptic area; MS: medial septum; VMN: ventromedial nucleus of the hypothalamus.

NTS PPG neurons from mGlu-Cre:tdRFP mice transduced by AAVs to express ChR2:eYFP showed strong GFP immunoreactivity (GFP-IR) in the plasma membrane of the cell bodies, dendrites and axons, which identified axons and terminals throughout the brain originating from the NTS PPG neurons. Analysis of hypothalamic projections of PPG-Cre neurons revealed a GFP-IR expression pattern indistinguishable from that seen with the YFP-PPG mice (compare Fig. 2B and C with Llewellyn-Smith et al. (2011)). This demonstrates that a significant proportion of the YFP axons in these regions originate from the NTS. For the current study we analyzed particularly GFP-IR over the rostrocaudal extent covering the BNST (Fig. 2). In concordance with the observed PPG axon distribution in the hypothalamus, there is also a strong similarity of NTS-PPG neuronal projections to BNST and surrounding areas with the YFP-PPG projections examined in Fig. 1. Whilst it is not possible for us to assign a percentage value when comparing the two sets of data (Fig. 1, Fig. 2), it is clear that the presence of NTS-PPG projections is substantial in the BNST.

### 3.2 Feeding experiments

#### 3.2.1 Dose responses

**Fig. 3:**
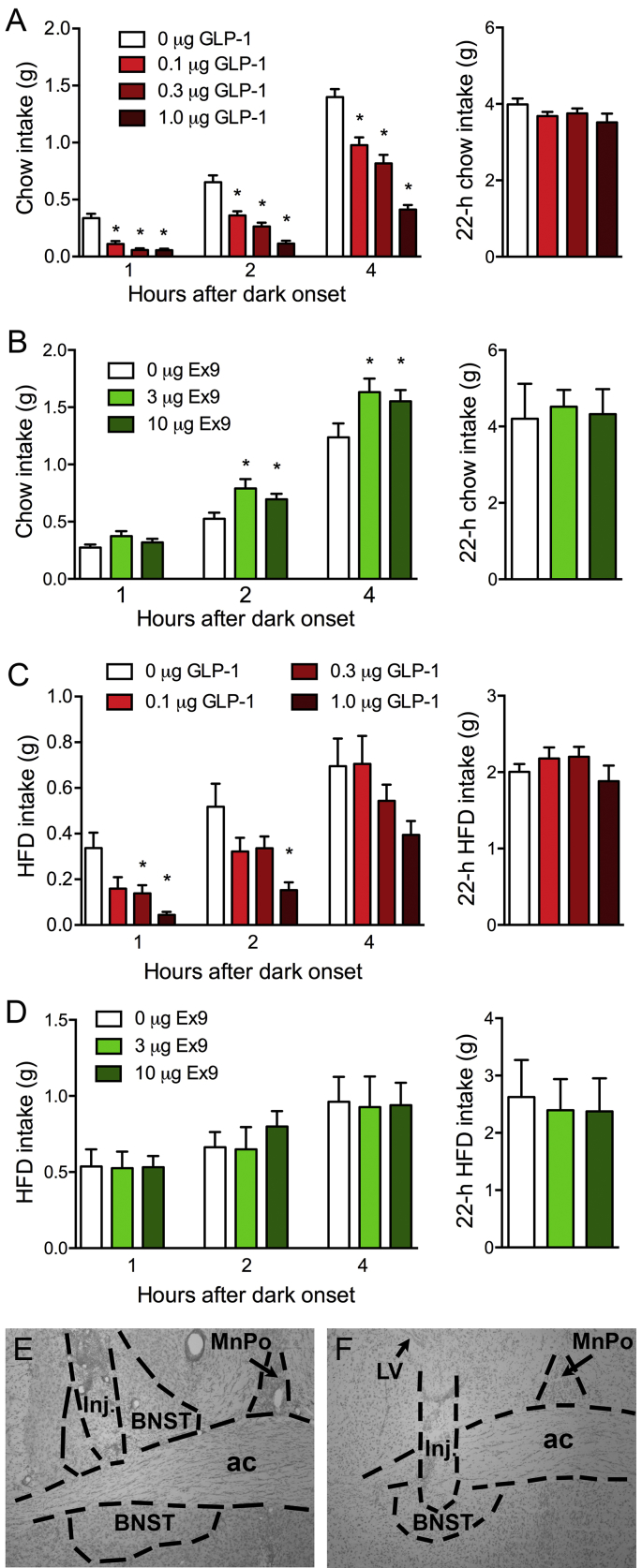
GLP-1R stimulation in the BNST suppresses food intake. (**A**) Chow intake after intra-BNST injection of GLP-1 is reduced in the first 4 h of the dark phase. (**B**) Intra-BNST injection of the GLP-1R antagonist Ex9 increases chow intake during the first 4 h of the dark phase. (**C**) In mice maintained on HFD, intra-BNST injection of GLP-1 suppresses feeding, but only up to 2 h after dark onset, and only at the higher doses. (**D**) HFD maintenance eliminates the feeding response to intra-BNST Ex9. (**E, F**) Representative images of a coronal sections from 2 different subjects showing BNST injection sites. Panel E shows an injection that was entirely within the dorsal BNST, and F shows an injection that extended into the ventral BNST. Inj. = injection damage. Data are means ± SEM, *p < 0.05 in Holm-Bonferroni comparison with vehicle conditions at their respective timepoints.

Intra-BNST GLP-1 injection significantly suppressed chow intake at 1, 2, and 4 h after dark-onset (F's (3, 14) = 34.47, 35.73, 55.70, respectively, P's < 0.0001) (see Fig. 3A). At 1 h, all doses of GLP-1 (0.1, 0.3, and 1.0 μg) differed from vehicle (P's < 0.01), and at 2 and 4 h, the magnitude of effect was dose-related with all treatment conditions differing significantly from one another (P's < 0.05). There were no effects on overnight intake (Fig. 3A). In chow-maintained mice, BNST GLP-1R antagonist treatment tended to increase feeding at 1 h after dark onset, but this did not reach significance (F (2, 15) = 3.309, P = 0.055). Ex9 did significantly increase feeding in these subjects at 2 and 4 h after dark onset (F's (2, 15) = 7.84, 5.64, respectively, P's < 0.01), with both the 3 and 10 μg doses differing from vehicle (P's < 0.01) (Fig. 3B), with no effect on overnight intake (Fig. 3B). In separate groups of mice maintained on HFD instead of chow, intra-BNST GLP-1 significantly suppressed feeding at 1 and 2 h post-dark onset (F's (3, 10) = 7.55, 4.88, respectively, P's < 0.05), but not all doses were effective, and there were no effects at 4 h or overnight (Fig. 3C). At 1 h, only the 0.3 and 1.0 μg doses of GLP-1 suppressed intake relative to vehicle (P's < 0.05) and at 2 h, only the 1.0 μg dose was effective (P < 0.05). Intra-BNST Ex9 failed to affect intake at any time in mice maintained on HFD (Fig. 3D).

**Fig. 4:**
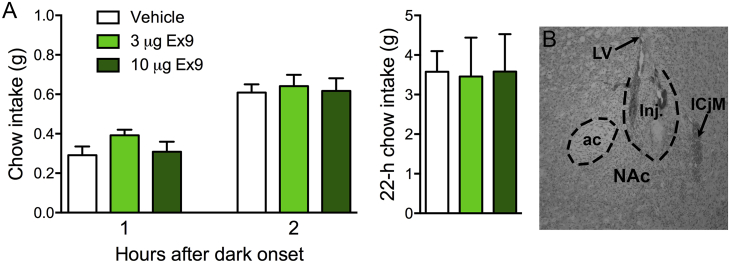
GLP-1R antagonist injected into the NAc has no effect on feeding. (A) Chow intake after intra-NAc injection of Ex9. (B) Representative image of a coronal section showing an NAc injection site. ICjM = Island of Calleja, Major island.

When Ex9 was delivered to the NAc, instead of the BNST, in chow-maintained mice, no significant effects on food intake were observed (Fig. 4).

#### 3.2.2 Stress-induced hypophagia

**Fig. 5:**
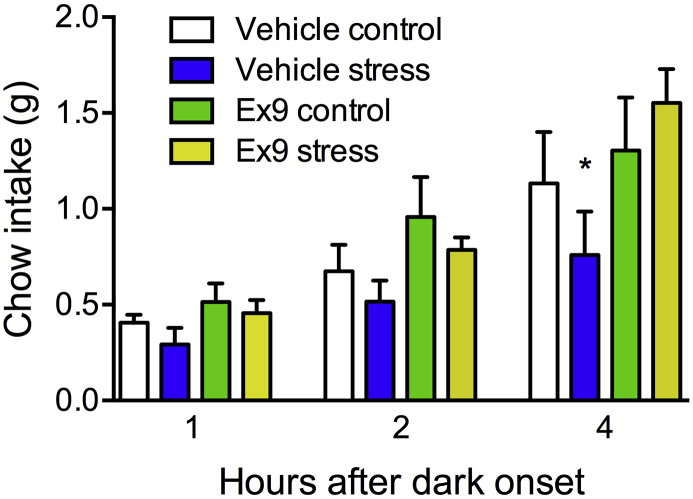
Acute restraint stress suppresses subsequent chow intake at 4 h after dark onset, and pretreatment with intra-BNST Ex9 attenuates this anorexic effect of stress. Data are means ± SEM, *p < 0.05 in Holm-Bonferroni comparisons with all other conditions at the 4 h timepoint.

The 30-min restraint stress tended to suppress intakes at all time-points measured, but a main effect of stress was observed only for overnight intake (F (1, 8) = 5.38, P < 0.05), and there were no pairwise significant differences. At 4 h after dark onset, there was a significant interaction between stress and Ex9 (F (1, 8) = 9.42, P < 0.05), where stress potently suppressed chow intake in the BNST vehicle group (P < 0.01) only, with no effect of stress in the Ex9 group (see Fig. 5). Intake in the Ex9 group after stress was significantly higher than that of the vehicle group (P < 0.05).

### 3.3 BNST electrophysiology

GLP-1R expressing BNST neurons were identified by their red fluorescence, and recordings were performed on cells both dorsal and ventral from the anterior commissure under optical control. No difference was observed in the response to GLP-1 or dopamine or in the basic electrical properties of GLP-1R neurons from these two locations. Similarly no differences were observed based on the sex of the animals used.

**Fig. 6:**
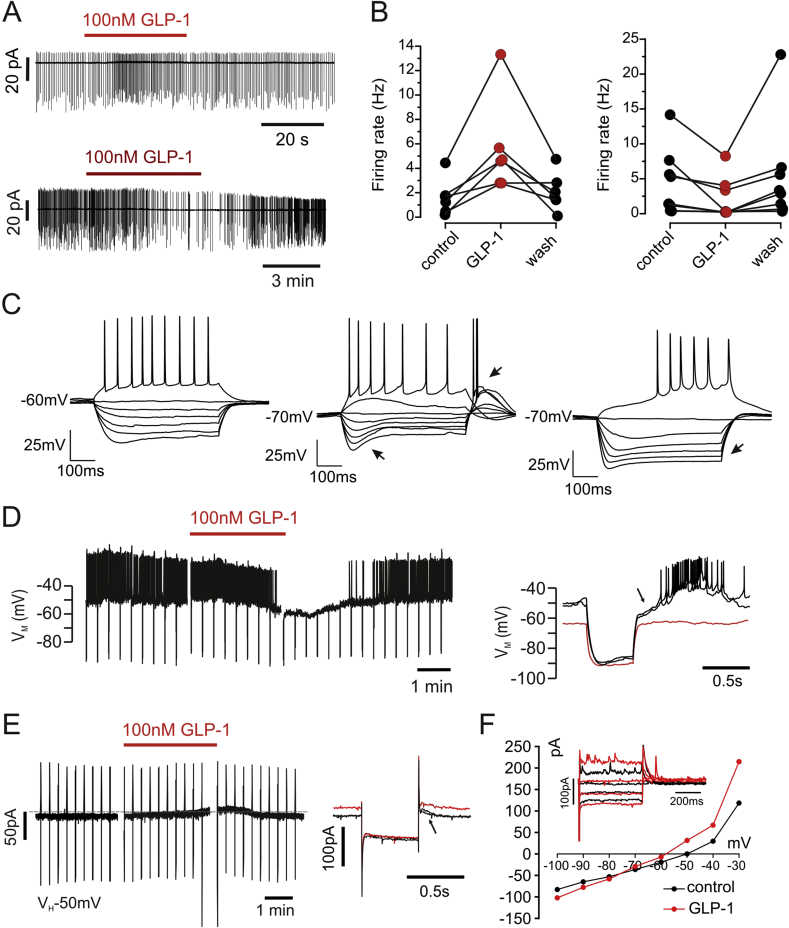
GLP1R expressing neurons display either excitatory or inhibitory responses to GLP-1. (**A**) Recordings made in the cell-attached configuration showed that GLP-1 causes either an increase or decrease in firing frequency of BNST GLP-1R neurons. (**B**) Mean firing rate of GLP-1 excited (left) and GLP-1 inhibited (right) BNST neurons before, during and after 100 nM GLP-1. (**C**) GLP-1R neurons fall into three groups. Type I (left) is characterized by sustained firing and little inward rectification. Type II (middle) is characterized by rebound spikes and h-type currents (arrows). Type 3 (right) is characterized by inward rectification (arrow). (**D**) Representative current clamp recording shows that in a neuron visually confirmed to express GLP-1R bath application of GLP-1 evoked a hyperpolarization leading to cessation of action potential firing. The response reverses after washout of GLP-1. The overlaid traces on the right demonstrate that the hyperpolarization under GLP-1 (red trace) is accompanied by an increase in membrane conductance. (**E**) The cell displayed in D recorded in voltage clamp at a holding potential of −50 mV. Bath-application of 100 nM GLP-1 elicits an outward current that reverses upon washout of GLP-1. Downward deflections in left hand traces in D and E indicate 500 ms current and voltage injections, respectively. Individual responses to these injections are shows on the right, with control traces in black and GLP-1 traces in red. Note outward current at −50 mV, but lack of current at −90 mV. Arrows in D and E indicate A-current. (**F**). Current-voltage relationship (IV) for a hyperpolarizing cell. The inset shows the current response to voltage steps to −90 mV, −70 mV, −50 mV and −30 mV under control (black) and in GLP-1 (red). (For interpretation of the references to colour in this figure legend, the reader is referred to the Web version of this article.)

In an initial set of experiments 100 nM GLP-1 was applied to the bath in a cell-attached loose patch configuration. GLP-1R neurons had a resting firing frequency of 3.3 ± 1.0 Hz, which either increased to 5.6 ± 1.6 Hz (n = 6, p < 0.01, paired *t*-test) or decreased to 2.1 ± 1.0 Hz (n = 8, p < 0.05, paired *t*-test) under GLP-1 (Fig. 6A and B). This was surprising, given that GLP-1R is considered to couple to Gs proteins, and thus excitatory responses only might have been expected. Consequently, a more detailed analysis of the electrical properties of these cells was performed.

Three different electrophysiological types of BNST neurons have been described previously in both rat and mouse (Daniel et al., 2017, Hammack et al., 2007) with type III the most abundant BNST neuron population in mouse and type II in rat (Daniel et al., 2017). Type II were characterized by rebound spiking or burst-firing, type III exhibited pronounced inward rectification, and type I were regular firing cells without the pronounced inward rectification of type III cells (Daniel et al., 2017, Rodriguez-Sierra et al., 2013). The neurons recorded here concurred with these published observations, however type II was the most abundant type amongst the GLP-1R neurons analyzed here. Altogether, 89 cells were classified; of these 34 were type 1, 41 type II and 14 type III (Fig. 6C).

Upon establishing the whole-cell configuration, cells were recorded in current clamp for a minimum of 5 min without any manipulation. Only cells that maintained a stable membrane potential over this period of time were further characterized. These cells exhibited a resting membrane potential of −60.2 ± 1.4 mV (n = 35) and an input resistance of 426 ± 44 MOhm. All recorded red fluorescent cells responded to bath application of 100 nM GLP-1, which caused a depolarization of 3.9 ± 0.5 mV (p < 0.001) in 11 of 28 cells tested and a hyperpolarization of 4.6 ± 1.0 mV (p < 0.001) in the remaining 17 (Fig. 6D). Both the depolarizing (4.3 ± 0.7 mV, n = 3, p < 0.05) and hyperpolarizing (4.2 ± 1.3 mV, n = 4, p < 0.01) responses persisted in the presence of 0.5 μM TTX, 10 μM DNQX and 25 μM APV, suggesting that they are due to direct postsynaptic effect of GLP-1 on the recorded cells.

In order to characterize the biophysical changes associated with the GLP-1 responses in more detail, voltage clamp recordings were performed. Cells were held at −50 mV and 100 nM GLP-1 was applied. Seven of the 13 cells recorded exhibited an outward current of 8 ± 4 pA (p < 0.05; Fig. 6E), whilst the remaining 6 cells exhibited an inward current of 6 ± 3 pA, which did not reach significance (p = 0.47). In three cells it was confirmed that the outward current at −50 mV was associated with an increase in conductance with a reversal potential close to −80 mV, indicative of the opening of a K^+^ channel (Fig. 6F).

**Fig. 7:**
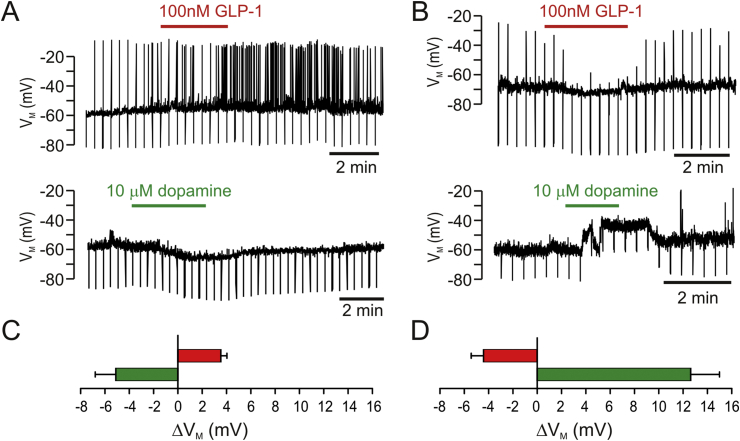
GLP-1R expressing BNST neurons respond to GLP-1 and dopamine in a reciprocal fashion. Representative current clamp recordings show that in neurons visually confirmed to express GLP-1R bath application of GLP-1 evoked either a small depolarization (**A**) or a small hyperpolarization (**B**). Cells that depolarized under GLP-1 exhibited a small hyperpolarization upon bath application of dopamine (bottom panel in **A**). Other neurons responded to bath application of GLP-1 by hyperpolarizing, and these cells depolarized under dopamine (**B**). Mean data for these two cell types is shown in **C** and **D**, respectively.

The BNST receives dopaminergic input from the VTA and the PAG acting on reward and stress pathways (Daniel and Rainnie, 2016, Kash, 2012). We next examined whether GLP-1R expressing cells in the BNST responded to dopamine (10 μM) (n = 8) and whether the dopamine response diverges between cells responding to GLP-1 differently (Fig. 7). In cells which had previously depolarized in response to GLP-1 we observed a hyperpolarization of 5.1 ± 1.7 mV (n = 5, p = 0.02). In cells which had previously hyperpolarized to GLP-1 we observed a depolarization of 13.2 ± 4.0 mV (n = 3, p < 0.05).

### 3.4 Projections from GLP-1R neurons in the BNST

**Fig. 8:**
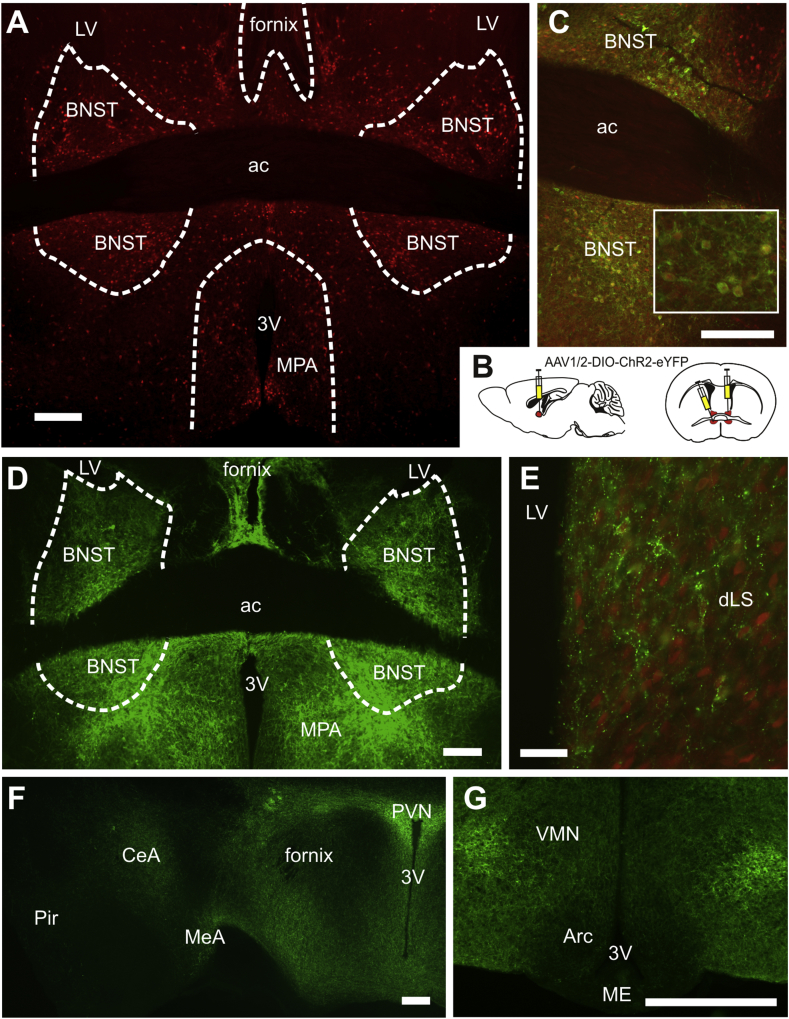
Virally-mediated cre-dependent tracing of BNST GLP1-R expressing neurons shows projections to target feeding circuits. tdRFP expression in GLP1-R cre mice reveal the presence of GLP1-R-expressing neurons within the BNST, dorsal and ventral to the anterior commissure (**A**). Cre expression in these neurons enabled GLP-1R-specific tracing by bilateral injections of AAV, dorsal and ventral to the anterior commissure (ac; **B**). Clear co-localisation of ChR2 eYFP and GLP-1R RFP expression was observed within the BNST, see inset for cytoplasmic RFP expression (red) and membrane-bound ChR2 eYFP (green) (**C**). ChR2 eYFP expression was strong within the BNST and could be seen coursing through the fornix (**D**). Within the dorsal lateral septum (dLS) ChR2:EYFP positive axons could be observed along the border of the lateral ventricle (LV) in close association with neurons expressing GLP-1R (**E**). Moving further from the BNST, EYFP containing axons could be observed within the paraventricular nucleus (PVN), the amygdaloid complex (**F**), and also the ventromedial hypothalamus (VMN; **G**). All scale bars equal 200 μm. 3 V: third ventricle; Arc: arcuate nucleus; CeA: central amygdaloid nucleus; LV: lateral ventricle; ME: median eminence; MeA: medial amygdaloid nucleus; MPA: medial preoptic area; MS: medial septum; Pir: piriform cortex. (For interpretation of the references to colour in this figure legend the reader is referred to the Web version of this article.)

Here we confirm our previous reports of a high density of GLP-1R cells in the BNST (Cork et al., 2015) (Fig. 8A & C). The projection targets of these cells were determined with a Cre-dependent AAV encoding ChR2:EYFP (Fig. 8D–G). Injection of 200 nl of AAV-ChR2:EYFP into the BNST of GLP-1R-CRE:tdRFP mice at Bregma +0.25 mm led to EYFP expression in tdRFP+ cell bodies from Bregma +0.50 mm to −0.60 mm, confirming that only cells expressing cre-recombinase enabled EYFP expression (Fig. 8C). EYFP+ cell bodies were found primarily in the BNST subnuclei, but also some in the medial septum (MS) and the ventral part of the LS (Fig. 8C, D). At Bregma −0.60 mm EYFP+ cell bodies were observed in the BNST nuclei around the fornix as well as the neighboring anterior hypothalamic area and the peduncular part of the lateral hypothalamus. All areas with EYFP+ cell bodies also exhibited a dense network of dendrites and non-varicose axons.

Varicose axons extended up to approximately 2.00 mm anterior from Bregma. No varicose axons were observed dorsal to the rhinal fissure, few were seen in the piriform cortex, and moderate numbers were found along the midline. More caudally, varicose axons along the midline persisted, being found in the infralimbic cortex and prelimbic cortex, as well as in the NAcC with increased density in its ventral part. Few axons were seen close to the ventral surface, and occasional axons in the piriform cortex. Innervation density increased at Bregma +1.40 mm, with dense innervation of both the dorsal and intermediate LS (Fig. 8E) as well as the NAcS. Moderate innervation occurred along the midline both dorsal and ventral from these nuclei, and few GFP-IR axons were found in the piriform cortex, as well as in the NAcC. More caudally (Bregma 1.10 mm–0.86 mm), piriform innervation was lost, but innervation of LS and NAcS increased. In contrast, there were few varicose axons in the medial septal nucleus and the NAcC. No axons were observed lateral from the ventricles or dorsal to the corpus callosum. Dense innervation with varicose axons was observed in the OVLT and the median preoptic nucleus with moderate innervation of the lateral part of the preoptic area.

Caudal from the BNST, at Bregma −0.60 mm, the subfornical organ and the anterodorsal thalamic nucleus were densely innervated. From here onwards a moderate density of varicose axons was seen in the paraventricular thalamic nucleus with few axons running down to the PVN which exhibited a high density of varicose axons (Fig. 8F). Similarly, many varicose axons were found in the nuclei of the central amygdala, but virtually no axons in the basolateral amygdala and piriform cortex (Fig. 8F).

No innervation was observed in the rostral hippocampus or the cortex, but there was moderate density of varicose axons in the thalamus just below the dorsal third ventricle, running towards the third ventricle, where they fanned out laterally. Dense innervation was found in the dorsomedial hypothalamus except the area directly adjacent to the third ventricle (Fig. 8G). A moderate density of varicose axons was seen throughout the hypothalamus, with notably less innervation of ARC and median eminence (Fig. 8G). At the level of the aqueduct many varicose axons were observed in the periaqueductal gray, from there moderate numbers of axons project ventrally along the midline and spread from the fasciculus retroflexus towards the medial lemniscus. Some axons were found in the rostral VTA and dense innervation occurred around the dorsal border of the retromammillary nucleus, with the nucleus itself and the other mammillary nuclei devoid of innervation. At Bregma −3.5 mm, dense innervation with varicose axons was seen in the interfascular nucleus, the parainterfascular nucleus and the paranigral nucleus of the VTA, but not in the interpendicular nucleus. Similarly, more lateral axons extended up to the medial lemniscus, but not to the substantia nigra, which was devoid of projections. Moderate numbers of varicose axons were observed along the midline up to the aqueduct and in the mesencephalic reticular formation. Innervation was dense in the lateral, and moderate in the dorsomedial, periaqueductal gray. Moderate numbers of varicose axons were observed in the amygdalohippocampal area. Sections more caudal than the aqueduct were not systematically analyzed. Projections to the NTS were present, but sparse.

## 4 Discussion

Our data show that injection of exogenous GLP-1 directly into the BNST potently reduces food intake in mouse, suggesting the BNST is a potential target for the weight-reduction effects of GLP-1 analogues. We also establish that blocking GLP-1 receptors in the BNST by microinjection of the GLP-1R antagonist Ex9 significantly increases acute food intake, verifying BNST GLP-1Rs as physiologically relevant target for endogenous GLP-1. This is further supported by our data showing that NTS PPG neurons, which constitute the most likely source of GLP-1 within the CNS, project strongly to the BNST. Furthermore, we found that GLP-1 elicits both excitatory and inhibitory electrical responses at cellular level within the BNST. This suggests that food intake effects of GLP-1 within the BNST are obtained through action on both orexigenic and anorexigenic pathways. Finally, the projection pattern of GLP-1R expressing cells in the BNST was established, in order to gain some insight into the downstream signaling pathways.

### 4.1 Exogenous and endogenous GLP-1 in the BNST affects food intake

Our data support a role for BNST GLP-1R in the physiological control of food intake, and suggest that this site may contribute to the feeding-suppressive effects of peripheral long-acting GLP-1R agonist treatments. Blockade of BNST GLP-1R increased feeding in non-stressed mice maintained on chow diet, suggesting that tonic endogenous activation of these receptors functions to limit feeding under normal conditions. We also examined the potential contribution of GLP-1R in the NAc, which has been previously identified as a region at which GLP-1R stimulation suppresses feeding in the rat (Alhadeff et al., 2012, Dossat et al., 2011). In the mouse, we found that delivery of the same doses of Ex9 that increased intake when delivered to the BNST failed to affect chow intake when injected into the NAc. This is consistent with the relatively sparse PPG innervation of the NAc observed here, and suggests that brain areas such as the BNST and others that receive a higher density of PPG input may be more important for the control of feeding in the mouse.

In addition, BNST GLP-1R blockade attenuated stress-induced hypophagia in the mouse. This is consistent with findings in the rat suggesting that GLP-1 input to the BNST may be activated by acute stress (Maniscalco et al., 2015). It is worth noting that in this experiment, we did not obtain a significant increase in chow intake after intra-BNST injection of Ex9 in the no-stress condition. We suggest that this is due to design differences between our stress experiment and the dose response experiment. In the restraint stress experiment, the vehicle vs Ex9 comparison is between-subjects, with both groups of a lower n than the entirely within-subjects dose response experiment, and more variability in intake across subjects. Therefore, we have lower power to detect a difference between those two conditions in the stress study, despite the trend toward increased intake with Ex9. However, the focus of this experiment is on the hypophagic effect of stress, and we see in these data that mice that received BNST Ex9 did not exhibit the restraint-induced anorexia that is observed in the vehicle-injected control group. Interestingly, PVN knockdown of GLP-1R revealed that these receptors are critical for a number of physiological responses to stress, though stress-induced hypophagia was not measured in that paper (Ghosal et al., 2017). Together these data highlight the diversity of the neural circuitry involved in the brain's response to stress. Based on our results, we conclude that endogenous GLP-1 action in the BNST is involved in homeostatic feeding control under non-stressed conditions, as well as in the anorexic response to acute stress.

When mice were maintained on HFD, the effects of BNST GLP-1 and Ex9 treatment were not as robust as on chow-fed mice. The duration of the GLP-1 effect was shorter, and higher doses were required to elicit measureable effects. Blockade of BNST GLP-1R failed to influence feeding at all in the HFD-maintained mice. Given the effects of pharmacological GLP-1R stimulation here, we can conclude that HFD does not eliminate GLP-1R expression or function in the BNST. However, the lack of Ex9 effect suggests that endogenous GLP-1 release in the BNST may be reduced by HFD, and the reduced effectiveness of GLP-1 itself raises the possibility that BNST GLP-1R have reduced sensitivity or signaling capacity under these conditions. It is possible that HFD decreases, but does not eliminate, GLP-1R expression or availability in BNST cells, or that HFD reduces GLP-1's binding affinity or efficacy at the receptor. Whatever the mechanism, the impaired response to GLP-1 in the BNST under HFD conditions may contribute to the overconsumption and weight gain observed.

### 4.2 PPG neuron projections to the BNST

The most likely source of the endogenous GLP-1 reaching the BNST is the PPG neurons of the lower brainstem. Indeed, visualisation of YFP immunoreactivity in the mesolimbic system in YFP-PPG mice demonstrated substantial innervation of the BNST with varicose PPG axons. This is consistent with previous reports of GLP-1R mRNA expression (Merchenthaler et al., 1999), BNST GLP-1 specific immunoreactivity in rat (Gu et al., 2013), GLP-1R promoter activity in mouse (Cork et al., 2015) and BNST specific, ICV delivered, GLP-1-dependant activation of c-fos (Gu et al., 2013, Rowland et al., 1997). In contrast, innervation of the NAc shell was low, and very few PPG axons were actually seen within the nucleus itself. Similarly, few PPG axons were seen in the LS. Larger numbers of axons were observed in the VTA. Previous studies in rat have demonstrated intake-suppressive effects of endogenous GLP-1 in the NAc and VTA (Alhadeff et al., 2012, Dossat et al., 2011), indicating that modest innervation might be physiologically relevant. This would suggest that the BNST innervation observed here is of significant importance.

Whilst it might be a reasonable assumption that the innervation of the BNST stems from the PPG neuron cell bodies located in the NTS of the lower brainstem, we verified this by targeting the NTS of mGlu-Cre:tdRFP mice with cre-dependent AAVs encoding ChR2-eGFP. These experiments revealed a virtually identical pattern of GFP-IR axons in the BNST to that observed with the YFP-PPG mouse, thus demonstrating that these axons indeed originate from the PPG neurons in the NTS. It remains to be determined functionally in future experiments that these PPG axons release the GLP-1 acting on the BNST neurons, though we consider this most likely.

### 4.3 Electrophysiological responses

GLP-1R-expressing cells in the BNST were located both dorsal and ventral from the anterior commissure and their electrophysiological fingerprint was rather variable, with individual GLP-1R cells corresponding to the previously defined electrophysiological cell types I to III. Whilst type II cells make up the largest fraction of GLP-1R cells, type I and III also contributed, with types I and II more abundant than reported for molecularly undefined BNST neurons in mouse, but similar to the distribution observed in rat (Daniel et al., 2017, Rodriguez-Sierra et al., 2013). Cellular effects of GLP-1 within the BNST were both excitatory and inhibitory. However, this neither correlated with the electrophysiological cell type, nor with the dorsal or ventral location of the individual cell within the BNST. Given that GLP-1R is considered to couple to G_s_ protein (Fletcher et al., 2016) it could be seen as surprising that inhibitory electrical responses were observed. However, at least in recombinant systems, GLP-1R has been demonstrated to be capable of signaling through G_q_ and G_i_ (Montrose-Rafizadeh et al., 1999). Additionally, a recent study investigating electrophysiological responses to GLP-1 in the paraventricular thalamic nucleus (PVT) reported inhibitory responses to GLP-1 that were partially cell autonomous and partially presynaptic (Ong et al., 2017). Furthermore, we have previously observed in hippocampus that GLP-1R neurons exhibit both excitatory and inhibitory responses upon GLP-1 exposure, and that the inhibitory hyperpolarizing response was caused by the activation of a K^+^ current (Cork et al., 2015). This latter observation was confirmed for some of the hyperpolarizing BNST neurons in the present study, although, like in Ong et al. (2017), in a number of cells the amplitude of the response was too small to allow definitive detection of a K^+^ current mediated effect. Independent of the ionic identity of the GLP-1 evoked current, our findings and those of Ong and colleagues might indicate that in neurons, coupling to G_s_ is not obligatory for GLP-1R.

The observation that similar proportions of BNST cells responded with excitation and inhibition in both cell-attached as well as whole-cell recordings increases the confidence that this is a physiologically relevant response. Given that the physiological outcome of GLP-1 application within the BNST was always anorectic, it would suggest that GLP-1 can act on both orexigenic and anorexigenic circuitry, inhibiting the former and activating the latter. The observation that cells inhibited by GLP-1 were activated by dopamine, and cells activated by GLP-1 were inhibited by dopamine further supports the hypothesis that GLP-1 acts differentially on two opposing cellular circuits within the BNST. Furthermore, the fact that within the BNST, GLP-1 and dopamine act on the same cells, suggests that orexigenic and anorexigenic pathways merge at this level. GLP-1 is clearly defined as a satiety peptide whilst enhanced striatal dopamine release has been linked to the activation of a hedonic orexigenic ‘appetition’ pathway (Han et al., 2016, Shechter and Schwartz, 2016). In that respect our finding that the electrical responses to dopamine and GLP-1 were the opposite in individual cells seems rather appropriate.

Whilst it is unclear whether the dopamine pathway thought to be involved in hedonic food seeking extends to the BNST, it is well established that the BNST receives strong dopaminergic input (Freedman and Cassell, 1994). Electrophysiological analysis found that only a small proportion of unidentified BNST neurons exhibited postsynaptic effects upon dopamine application (Kash et al., 2008). A second electrophysiological study selectively recording from corticotropin-releasing factor (CRF) neurons revealed that these neurons are depolarized by dopamine (Silberman et al., 2013). It is unclear whether the depolarizing subgroup of GLP-1R cells recorded in our study corresponds to these CRF neurons. No previous electrophysiological study has reported a hyperpolarizing response to dopamine in the BNST.

The BNST has been implicated in the integration of stress and reward behavior, as well as in both anxiogenic and anxiolytic effects and both inhibition and stimulation of food intake (Betley et al., 2013, Kash et al., 2015). Given this dichotomy in various aspects of behavior, it might not be surprising to observe these two opposite electrical effects to GLP-1 as well as dopamine.

### 4.4 BNST projections

The BNST has extensive connectivity with the brainstem, hypothalamic and limbic structures, and is recognized for its corresponding role in autonomic, neuroendocrine and behavioral functions (Crestani et al., 2013). Most notably, it is known for processing reward/addiction and stress/anxiety, but has recently been shown to have an additional role in appetitive responses. This model of BNST circuitry suggests it to be an important site of hedonic and homeostatic signal integration. Arguably the most comprehensive analysis of BNST projections has been performed in rat (Dong and Swanson, 2004a, Dong and Swanson, 2004b). These studies analyzed projections from the various BNST subnuclei, but not of specific celltypes within the BNST. In contrast, our study only labeled projections from those BNST neurons that express GLP-1R. Comparison of the projection pattern obtained by us with that from rat revealed a largely overlapping pattern and suggests that the BNST GLP-1R neurons are a subpopulation of BNST cells that is representative for the entire population, rather than a subgroup that only projects to few select targets. Consequently, it might be expected that GLP-1 can influence the majority, if not all, of the physiological functions assigned to BNST neurons. This conclusion is made, however, under the presumption that rat and mouse projection patterns of BNST neurons do not differ significantly.

Electrophysiology and tracing studies have shown it receives glutamatergic inputs from the hippocampus and medial prefrontal cortex, and in turn projects to dopamine neurons in the VTA (Jalabert et al., 2009). The BNST is also thought to send GABAergic inhibitory projections to the lateral hypothalamus (LH), which suppresses LH glutamatergic neurons to promote feeding. Optogenetic activation of this pathway induced voracious feeding, reinforced motivational behaviours for subsequent light stimulation, and increased preference for high-fat food (Jennings et al., 2013). Conversely, photoinhibition suppressed feeding in starved mice, and triggered avoidance behaviour (Jennings et al., 2013).

We found that BNST GLP-1R-expressing cells project to many established BNST target sites that have known roles in the control of energy balance and stress responses, including among others the LS, PVN, dorsomedial hypothalamus, and CeA. Notably, we observed a high density of BNST GLP-1R cell fibers in regions that are themselves known to express GLP-1R, and in some cases are established locations for GLP-1R action on behavior and physiology. For example, the PVN displayed heavy innervation in this study, and GLP-1R in the PVN have recently been shown to play a mediatory role in several aspects of the stress response (Ghosal et al., 2017). As can be seen in Fig. 8E, the LS receives projections from these BNST GLP-1R cells, and many cells within the LS also express GLP-1R. The dorsal LS, in particular, is a site where endogenous GLP-1 action suppresses food intake and motivation for palatable food in the rat (Terrill et al., 2016). The VTA is another such example, receiving input from the BNST GLP-1R neurons in our study, and established as a site of action for GLP-1's suppressive effects on feeding and cocaine self-administration in the rat (Alhadeff et al., 2012, Schmidt et al., 2016). Many of these brain regions, in turn, project reciprocally to the BNST, though we do not know whether those projections include axons from GLP-1R cells in those locations.

Collectively, these results indicate the vital role of afferent projections to, and efferent projections from the BNST in a critical feeding circuitry, and our results suggest that GLP-1 plays a significant part in this circuit.

## 5 Conclusions

This study reveals further complexity in the central GLP-1 circuitry than previously assumed, with interconnections among the brain regions and cells that receive endogenous GLP-1 input from the hindbrain PPG neurons as well as a mixture of excitatory and inhibitory responses at cellular level. Such an arrangement could function to coordinate a neuroanatomically diverse network to execute multi-faceted behavioral and physiological responses, such as feeding, stress and anxiety, motivation and reward seeking behaviors.

## Author contributions

DLW and ST conceived the project. NAL performed the *in vivo* food intake studies. IJE performed the electrophysiology. PY performed the immunocytochemistry on PPG mice, JER performed the stereotaxic injections and immunocytochemistry for cre-dependent tracing. All authors analyzed the data. ST and DLW wrote the manuscript, and all authors contributed to editing and provided intellectual input.
